# Serum profiling of the antibody response to HPV in women with or without abnormal cervical cytology undergoing cervical cancer screening

**DOI:** 10.3389/fimmu.2025.1612761

**Published:** 2025-07-31

**Authors:** Radwa Ewaisha, Mack T. Ruffin, Stacy Williams, Yunro Chung, Cecilia R. DeGraffinreid, Electra D. Paskett, Paul L. Reiter, Ji Qiu, Dean E. Brenner, Karen S. Anderson

**Affiliations:** ^1^ Department of Microbiology and Immunology, Faculty of Pharmacy, Alexandria University, Alexandria, Egypt; ^2^ Department of Family and Community Medicine, Penn State College of Medicine, Hershey, PA, United States; ^3^ Center for Personalized Diagnostics, Biodesign Institute, Arizona State University, Tempe, AZ, United States; ^4^ College of Health Solutions, Arizona State University, Phoenix, AZ, United States; ^5^ Division of Population Health Sciences, Ohio State University Comprehensive Cancer Center, Columbus, OH, United States; ^6^ Division of Cancer Prevention and Control, Department of Internal Medicine, College of Medicine, The Ohio State University, Columbus, OH, United States; ^7^ Division of Health Behavior and Health Promotion, College of Public Health, The Ohio State University, Columbus, OH, United States; ^8^ Department of Pharmacology, University of Michigan Medical School, Ann Arbor, MI, United States

**Keywords:** antibodies, HPV, cervical cancer, cervical intraepithelial neoplasia, NAPPA, protein microarrays, serology, early detection antibodies

## Abstract

**Introduction:**

Understanding the humoral immune response to HPV is important for understanding the natural history of infection and developing biomarkers for early detection of cervical cancer. This has been technically limited by HPV type diversity and challenges of high-throughput protein expression and display. This study aimed to profile the humoral immune response to the proteomes of 12 HPV types in women with or without abnormal cervical cytology undergoing cervical cancer screening.

**Methods:**

To detect serum antibodies (Abs) against HPV, we developed custom HPV high-density diffusion-free nucleic acid programmable protein arrays (HD-NAPPA) displaying the proteomes of 2 low-risk (HPV6 and 11) and 10 high-risk (HR) HPV types (HPV16, 18, 31, 33, 35, 39, 45, 51, 52 and 58). Arrays were probed with sera from women undergoing screening for cervical cancer, with normal (n=82) or abnormal (n=54) cervical cytology. HPV DNA testing and typing were done on cytology samples from all participants using an assay that detects 37 HPV types.

**Results:**

Abs to any HPV protein were detected in 47.6% (95% C.I.: 36.5-58.8%) and 40.7% (95% C.I.: 27.9-54.9%) of women with normal and abnormal cytology, respectively and in 44.9% (95% C.I.: 36.4-53.6%) of all women. HPV16 DNA was the most frequently detected type (36.8%, 95% C.I.: 27.4-47.4%), however, Abs against HPV16 were remarkably the least frequently detected (7.4%, 95% C.I.: 3.8-13.5%). The most frequently detected Abs were against L1, in 30.1% (95% C.I.: 22.7-38.7%) of all women (31.7% and 27.8% of women with normal and abnormal Pap, respectively). Abs against E1 and E4 were the most (in 24.3%, 95% C.I.: 17.5-32.5%) and least (13.2%, 95% C.I.: 8.2-20.4%) frequently detected E-Abs in all women, respectively. Among all subjects with antibodies to either L1 or L2, 39.0% (95% C.I.: 24.6-55.5%) of those with L1 antibodies and 51.9% (95% C.I.: 32.4-70.8%) of those with L2 antibodies were positive for the antigen from only one HPV type.

**Conclusion:**

Our findings shed light on the kinetics of HPV-specific humoral immunity in women with normal or abnormal cervical cytology and highlight the need for comprehensive immune profiling in different health and disease stages.

## 1 Introduction

Human papillomavirus infection is a precursor event to cervical cancer (1), the fourth most common female malignancy worldwide (2). It is also associated with other types of cancers including anogenital and oropharyngeal cancers (3, 4). There were 660,000 new cases of cervical cancer worldwide in 2022 with an annual global mortality rate of 350,000 deaths (5, 6). Of more than 200 closely related HPV types, the vast majority of cervical cancer cases worldwide are attributable to 7 high-risk HPV types (HPV16, 18, 31, 35, 45, 52, and 58) (7, 8), with HPV16 and 18 being responsible for over 70% of cases (9).

High-grade lesions are clinically detectable and surgically removable, making cervical cancer a preventable disease (10). In developed countries, screening recommendations involve HPV nucleic acid testing or regular cytology (Pap smear) (11, 12). In low- and middle-income countries (LMICs), population based cervical cancer screening is limited mainly due to challenges of the implementation of regular screening, which include the high cost of cytology and nucleic acid testing (13) and sociocultural barriers in traditional societies that limit access to medical personnel during screening procedures (14).

The humoral immune response to HPV has been of interest for the development of biomarkers for early detection and selection of women for colposcopy (15, 16). Serology has also been pivotal for understanding the natural history of infection, pathogenesis, and vaccination efficacy (17–20). Understanding the humoral immune response in the settings of HPV infection and cancer requires proteome-wide immune profiling. This has been technically limited by challenges of high-throughput protein synthesis and display. Most studies reporting HPV-specific antibodies (Abs) have thus selected specific antigens from the most common HPV types (21–24). In the present study, to detect serum Abs against the proteomes of multiple HPV types, we used High Density Nucleic Acid Programmable Protein Arrays (HD-NAPPA) (25, 26). HD-NAPPA has enabled rapid profiling of the humoral immune response in diverse applications such as tuberculosis (27), type 1 diabetes (28), COVID-19 (29), and others (30, 31).

Here, we have developed HPV HD-NAPPA nanowell arrays displaying the proteomes of 2 low-risk and 10 high-risk HPV types. To better understand the humoral immune response in HPV infection and in different stages of cervical cancer pathogenesis, we have used these arrays to systematically investigate the serologic immune response to HPV in women with normal cervical cytology with or without detectable HPV DNA and in women with abnormal cervical cytology.

## 2 Methods

### 2.1 Sample selection

We used samples from the Community Awareness Resources and Education (CARE) project developed by the Ohio State University Center for Population Health and Health Disparities (CPHHD) funded by the National Institutes of Health (NIH) (32). Serum samples were collected from women scheduled for a routine Papanicolaou (Pap) smear test at 17 participating health clinics located throughout the Appalachian Ohio region between January 2006 and December 2008 (n=1131) (33). Appalachian Ohio lies in the southern and eastern parts of Ohio, comprising 32 counties. The area is known to have elevated levels of cancer incidence and mortality in contrast to non-Appalachian regions (34–36) and has been categorized as an underserved and unique demographic by the National Cancer Institute (NCI). For this study, a total of 136 serum samples were randomly selected to include 41 samples from each group and all 13 samples from women for which cervical intraepithelial neoplasia (CIN) grade is available. These included 82 samples from women with normal cervical cytology, of which 41 were positive for at least one of four high risk HPV types (HPV16, 18, 33, or 45), and 41 had no detectable HPV. The remaining samples (n=54) were from women with abnormal cervical cytology of which 13 underwent colposcopy and biopsy and for which CIN grade is available. The classification of abnormal cervical cytology was done according to the 2001 Bethesda System for Reporting Pap Smear Results (37). Cases with abnormal cervical cytology were classified as having atypical squamous cells of undetermined significance (ASC-US), atypical glandular cells (AGC), low-grade squamous intraepithelial lesions (LSIL), high-grade squamous intraepithelial lesions (HSIL), or carcinoma. Serum samples were collected using a standardized sample collection protocol and stored at -70°C until use. Written informed consent was obtained from all subjects under institutional review board approval. Age and race information were collected using a self-administered questionnaire prior to the Pap smear and following consent.

### 2.2 HPV DNA detection and typing

HPV DNA testing was done on all cytology samples (N=136) collected in specimen transport medium (Qiagen, Valencia, CA) after shipping to the Centers for Disease Control and Prevention (CDC) as previously described (37). Briefly, DNA was extracted from 150 µL of each sample using the MagNA Pure DNA kit III (Roche, Indiannapolis, IN). HPV typing was done using the Linear Array (LA) HPV Genotyping Assay (Roche, Indiannapolis, IN), which detects 37 HPV types (6, 11, 16, 18, 26, 31, 33, 35, 39, 40, 42, 45, 51, 52, 53, 54, 55, 56, 58, 59, 61, 62, 64, 66, 67, 68, 69, 70, 71, 72, 73, 81, 82, 83, 84, 89, and IS39). Because of known cross-reactivity between HPV33, 35, and 58 and the XR (52) probe, the presence of HPV52 was confirmed by an HPV52 quantitative PCR assay in XR-positive samples that are also positive for any of the cross-reactive HPV types (38).

### 2.3 Generation of custom HPV high-density microarrays

**Figure 1:**
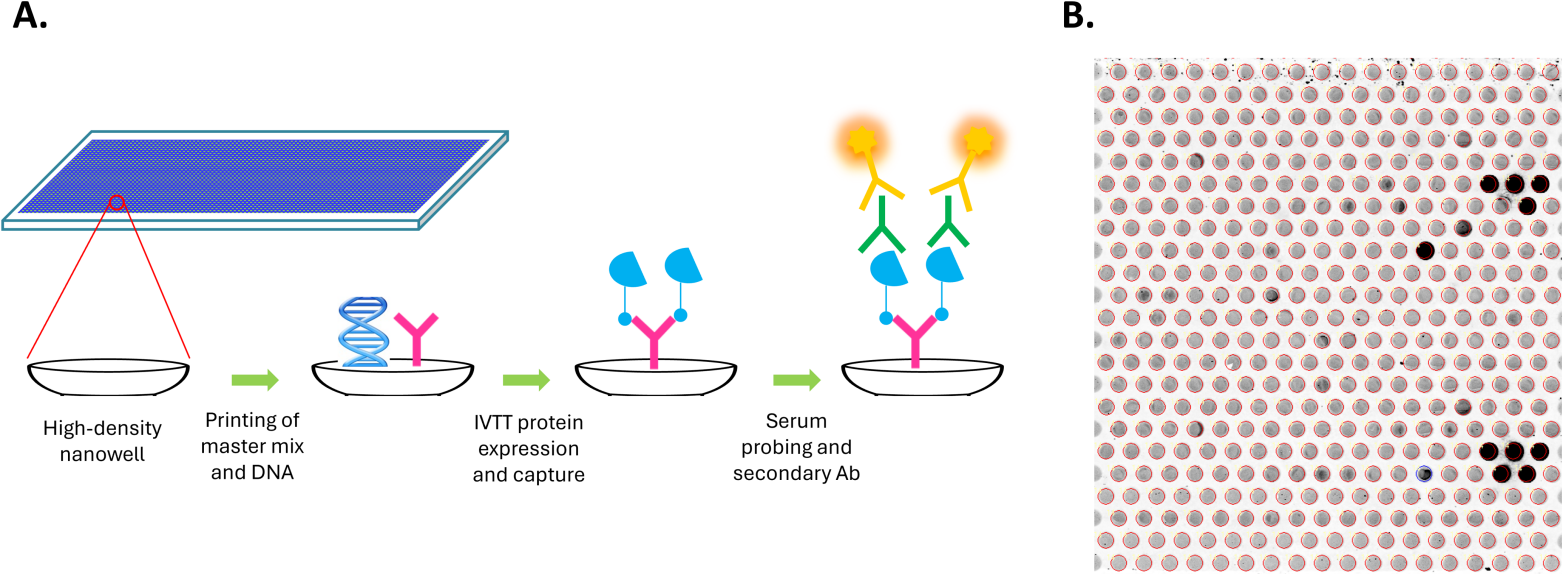
Generation of custom HPV high-density nanowell arrays. **(A)** cDNA plasmids encoding HPV or control antigens along with master mix containing anti-GST Ab were spotted in individual nanowells etched on the surface of glass slides. Antigens were then expressed as GST fusion proteins from cDNA plasmids using an *in vitro* transcription translation (IVTT) system and were immediately captured onto the anti-GST Abs in the nanowells. Nanowells are sealed during expression, minimizing protein diffusion to neighboring spots following expression. Arrays were then probed using patient serum and Alexa Fluor 647-labeled goat anti-human IgG secondary Ab was used for detection. **(B)** Detection of specific IgG Abs using the HPV HD nanowell arrays. Each nanowell contains a unique HPV protein or control protein. Darker nanowells indicate higher immunoreactivity.

Custom HPV high-density nucleic acid programmable protein arrays (HD-NAPPA) were produced as previously described (25, 31, 39) with modifications described here. HD-NAPPA is a silicon nanowell version of the NAPPA technology (40, 41) that allows high throughput and rapid *in situ* antigen expression and display for Ab detection in the settings of cancer (42–44), infectious diseases (45, 46), and autoimmunity (47, 48). In HD-NAPPA, cDNA plasmids encoding the antigens are spotted in individual nanowells that are then sealed, minimizing protein diffusion to neighboring spots following expression and enabling a higher throughput of more than 10,000 proteins per array (39) (**Figure 1**).

#### 2.3.1 DNA preparation

Genes encoding proteomes of 2 low risk (HPV6 and 11) and 10 high risk (HPV16, 18, 31, 33, 35, 39, 45, 51, 52, and 58) HPV types cloned into the T7-based mammalian expression vector pANT7_cGST previously described (49, 50) were used. For HPV16 E6, E7, and L1 the non-codon-optimized versions, which had higher protein expression, were used instead. All genes were sequence verified and are publicly available at https://dnasu.org/DNASU/ (51). Another set of 117 non-HPV genes cloned in pANT7_cGST were used as controls on all arrays (**Supplementary Table 1**). As positive controls, we used several genes encoding Epstein-Barr virus (EBV) antigens, a virus that will have infected over 95% of individuals by the time they reach adulthood (52). These include Epstein-Barr Nuclear Antigen (EBNA), small capsomere-interacting protein (BFRF3), and EBNA2. Genes from other viruses were also included, specifically H1N1 Nucleoprotein, H3N2 Nucleoprotein, and HCMV2 Viral transcription factor IE2 (UL122). Other positive controls used included purified mouse IgG, human IgG and human IgA at concentrations of 40–200 ng/L in printing master mix to control for the reactivity of secondary Abs (**Supplementary Table 1**; **Supplementary Figure 1**). Negative controls included 93 genes randomly selected from the DNASU plasmid repository (including the plasmid encoding the GST fusion protein) and printing master mix (MM) with no plasmid (**Supplementary Table 1**; **Supplementary Figure 2**). Negative controls were used for array signal intensity normalization and establishment of cut-off values. Plasmid DNA extraction and quality assurance were performed as previously described (29). DNA concentration was normalized to 100 ng/uL before printing.

#### 2.3.2 Piezoelectric printing

High-density nanowell arrays were produced at the Arizona State University Center for Solid State Electronics Research (CSSER) as previously described (39). HPV nanowell arrays were printed using an au302 piezoelectric dispensing system (Engineering Arts LLC, Tempe, AZ) through “on the fly” non-contact dispensing with a 16-pin dispensing head. Each nanowell was filled with 1,200 picoliters of printing mix followed by 300 picoliters of DNA. Each array was equally divided into 16 sub-arrays. HPV genes and positive control genes were each printed in duplicate in each sub-array. Following printing, arrays were stored desiccated in a nitrogen-filled container at room temperature until use.

### 2.4 Protein expression on the HPV nanowell arrays

SuperBlock (Thermo Fisher Scientific, Rockford, IL) was used to block the arrays before protein expression to reduce non-specific binding. Arrays were then rinsed with DI water and centrifuged before they were filled with human *In Vitro* Transcription and Translation (IVTT) coupled system (Thermo Fisher Scientific). The wells were sealed with a polystyrene membrane under a pressure of 200 PSI. Arrays were incubated in a custom reactor device at 30˚C for 2 hours for protein expression and at 15˚C for 30 minutes for protein capture by the anti-GST Ab. Wells were then blocked for 30 min with 5% skimmed milk in phosphate buffered saline with 0.2% tween-20 (PBS-T). For detection of protein expression levels, mouse anti-GST monoclonal Ab (Cell signaling technology, Danvers, MA) was added and Alexa Fluor 647-labeled goat anti-mouse IgG (H+L) secondary Ab was used for detection (Thermo Fisher Scientific).

### 2.5 Detection of serum Abs

HPV nanowell arrays were expressed on the day of the assay to be probed with serum samples. A custom 16-well gasket (GraceBio-Labs, Bend, OR) was used on each array to separate sub-arrays to allow the addition of a different serum sample to each one. Serum was diluted 1:100 in 5% skim milk in PBST and each individual serum sample was added to a sub-array. Arrays were incubated overnight at 4˚C with gentle shaking and then rinsed with 5% milk in PBS-T. Bound Abs were detected using Alexa-Fluor 647-conjugated goat anti-human IgG Ab (H-L). Arrays were rinsed to remove unbound secondary Ab and dried by centrifugation before scanning.

We determined if serum Abs were reactive to more than one homologous Ag from different HPV types (e.g. Abs in serum from one patient recognize the L1 Ag from multiple HPV types). This allows the detection of potential cross-reactivity of Abs to Ags with high sequence similarity. However, Ab reactivity to homologous Ags from multiple HPV types could be due to past exposure to a different HPV type or the presence of this different HPV type in a different site of the body such as the oropharynx. Seropositivity was determined using the same cutoff value used throughout the study (see statistical analysis below).

### 2.6 Protein array image analysis and quantification

Arrays were scanned at 635 nm with a Tecan PowerScanner (Tecan Group, Männedorf, Switzerland). Images were analyzed using the ArrayPro Analyzer software (MediaCybernetics, Bethesda, MD) for the quantification of signal intensity of individual spots. Normalization of raw intensity values was performed by dividing each spot signal intensity value by the median intensity of all spots to calculate the signal/background ratio.

### 2.7 Statistical analysis

The correlation of raw signal intensities of protein expression between the sub-arrays on an array randomly selected for quality control was determined with scatter plots and the Pearson correlation coefficient (R) was calculated to assess consistency. Protein expression on the arrays was measured by calculating the mean values of raw signal intensities of duplicate spots printed on the array. Mean values (of duplicate spots for a given Ag) of normalized signal intensity were calculated and a normalized signal cutoff of 1.5 was used to define seropositivity for any given Ag. Seropositivity rates were calculated for specific HPV proteins or specific types and their 95% confidence intervals were calculated. Pairwise comparisons of age differences among the four groups of women were conducted using the Dunn’s test (R version 4.5.0). A false discovery rate (FDR) adjusted p-value less than 0.05 was considered statistically significant. A total of 136 samples were run on the arrays.

The age distributions were not normally distributed. To account for skewed data, we used the pairwise comparison of the Wilcoxon-rank sum test, resulting in no significant age difference between the 4 groups of women (FDR adjusted p-value=0.05).

## 3 Results

### 3.1 Production and reproducibility of HPV HD-NAPPA protein arrays

**Figure 2:**
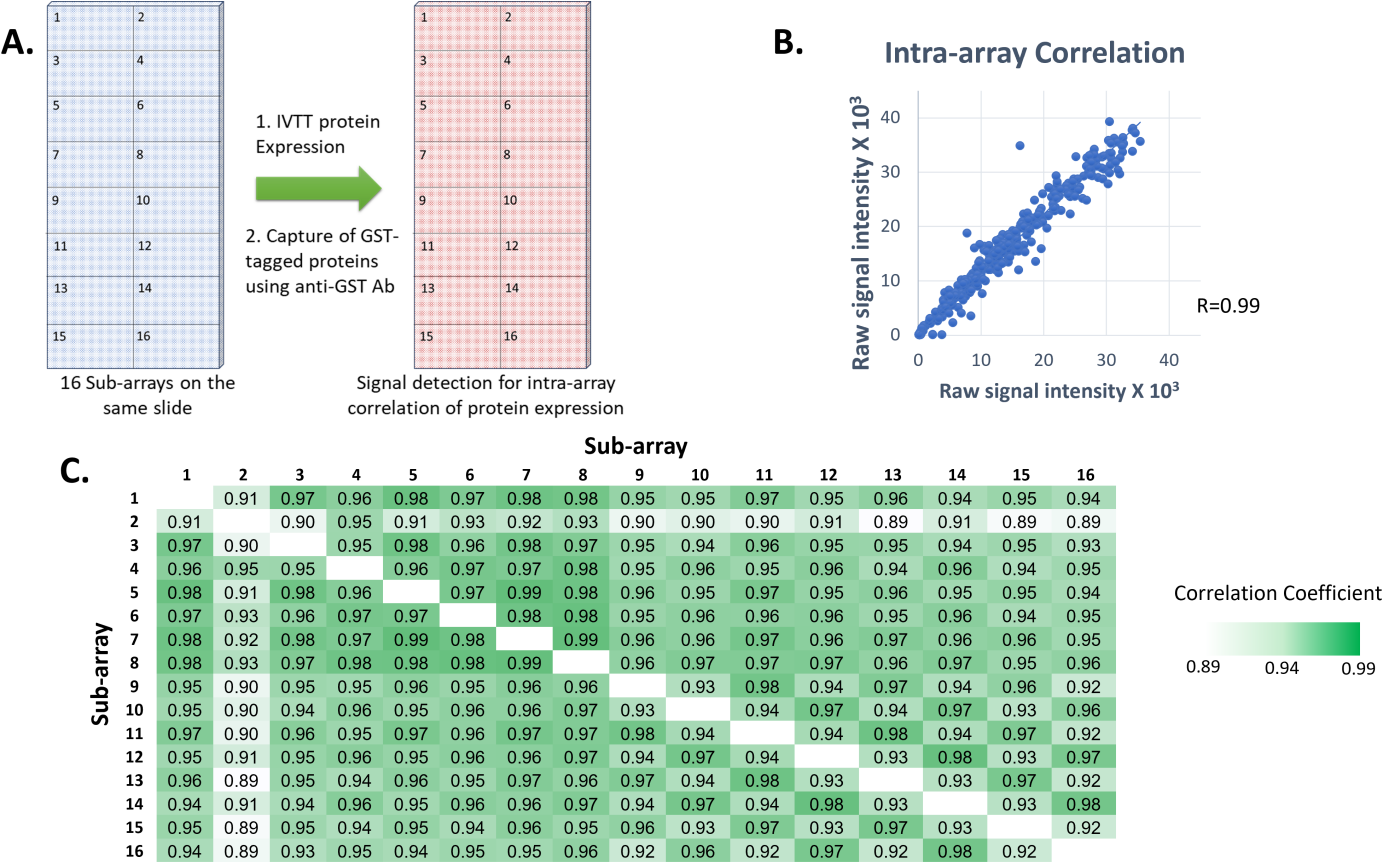
Reproducibility of printing and protein expression on our custom HPV high density microarrays. **(A)** Each printed slide contained 16 identical sub-arrays, each comprising the full set of 12 HPV genomes in duplicate plus control proteins. GST-tagged proteins were expressed from plasmids printed in all nanowells on each slide, an anti-GST monoclonal Ab was used and Alexa Fluor 647-labeled goat anti-mouse IgG (H+L) secondary Ab was used for detection of protein expression levels for calculation of intra-array correlation. **(B)** Correlation of protein signal intensities from all spots from two sub-arrays on the same slide following protein expression (R=0.99). **(C)** Correlation coefficient values between each two sub-arrays on a randomly selected slide following protein expression.

The quality and reproducibility of the HPV HD microarray printing was evaluated by DNA staining with picogreen and by measuring protein levels expressed and displayed using anti-GST monoclonal Ab (**Figure 2**). Sixteen identical sub-arrays were printed on each array. The correlation coefficients of anti-GST signals were determined for each two sub-arrays. They were found to be in the range of 0.89-0.99, with 85.4% of the calculated R values (n=120) ≥0.95 and 89.2% ≥0.93 (**Figure 2**), reflecting high correlation of protein display between the sub-arrays.

### 3.2 Characteristics of study samples

In this study, we aimed to determine the frequency and specificity of HPV-specific Ab responses in women with or without an abnormal Pap result. Age, race, HPV DNA status, and number of HPV types detected in patients contributing samples to the study are shown in **Table 1**. The participating health clinics had a high proportion of white patients. The racial distribution of samples was random. There was no significant age difference between the four groups of women (minimum Benjamini-Hochberg FDR-adjusted p-value=0.06 among the pairwise comparisons). Of 82 women with normal cervical cytology, 41 women had no detectable HPV and 41 women had at least one of the 4 HR HPV types, HPV16, 18, 33, or 45. These three are the most common types implicated in cervical cancer (53, 54).

**Table 1:** Characteristics of study samples.

Characteristics	Disease status				Total
	Normal Pap (N=82)		Abnormal Pap (N=54)		
	With no detectable HPV	With any of 4 HR HPV types*	With ASCUS, no CIN	Referred for colposcopy and biopsy	
N	41 (30.1%)	41 (30.1%)	41 (30.1%)	13 (9.6%)	136
White	39 (95.1%)	40 (97.6%)	38 (92.7%)	13 (100%)	130 (95.6%)
Other	2 (4.9%)	1 (2.4%)	3 (7.3%)	0 (0%)	6 (4.4%)
Age Mean	36.6	28	33	35.1	32.8
Age ≤ 30	19 (46.3%)	31 (75.6%)	21 (51.2%)	7 (53.8%)	78 (57.4%)
Age >30	22 (53.7%)	10 (24.4%)	20 (48.8%)	6 (46.2%)	58 (42.6%)
Number of HPV types					Total (N=95)
0	41 (100%)	0 (0.0%)	19 (46.3%)	2 (15.4%)	21 (15.4%)
1		15 (36.6%)	10 (24.4%)	5 (38.5%)	30 (22.1%)
2		11 (26.8%)	8 (19.5%)	3 (23.1%)	22 (16.2%)
3		7 (17.1%)	1 (2.4%)	1 (7.7%)	9 (6.6%)
4		5 (12.2%)	3 (7.3%)	1 (7.7%)	9 (6.6%)
5+		3 (7.3%)	0 (0%)	1 (7.7%)	4 (4.2%)

*HPV 16, 18, 33, or 45.

Among women who had been referred for colposcopy and biopsy (n=13), four had atypical squamous cells of undetermined significance (ASCUS), one had atypical glandular cells (AGC), three had low-grade squamous intraepithelial lesion (LGSIL), four had high-grade squamous intraepithelial lesion (HGSIL), and one had squamous cell carcinoma (**Table 2**). In this group, the number of women with CIN grades I, II, and III were 4, 5, and 4, respectively (**Table 2**).

**Table 2:** CIN grade and cervical cytology of women with abnormal Pap who underwent colposcopy and biopsy.

CIN grade	Number of women (N=13)		
	CIN I (N=4)	CIN II (N=5)	CIN III (N=4)
ASCUS	2	2	0
AGC	0	0	1
LGSIL	1	2	0
HGSIL	1	1	2
Squamous cell carcinoma	N/A	N/A	1

AGC, atypical glandular cells; ASCUS, atypical squamous cells of undetermined significance; LGSIL, low-grade squamous intraepithelial lesion; HGSIL, high-grade squamous intraepithelial lesion.

The introduction of HPV vaccination occurred in 2006 and was approved for use in females aged 9-26. A significant proportion of women in this study (42.6%) were above the age of 30 (**Table 1**). HPV vaccination status was known for only less than 20% of women participating in the CARE study (n=1131), preventing us from including this variable in our analyses. Nonetheless, among women with available vaccination data, less than 6% had received any doses of the HPV vaccine, indicating that the impact of vaccination on our findings was probably insignificant (33).

### 3.3 HPV DNA detection and typing in cervical cytology samples

HPV DNA was detected in samples from cervical cytology and typed using the LA HPV Genotyping Assay which detects 37 HPV types. These include 23 high-risk (HR; 16, 18, 26, 31, 33, 35, 39, 45, 51, 52, 53, 56, 58, 59, 64, 66, 67, 68, 69, 70, 73, 82, and IS39) and 14 low-risk (LR; 6, 11, 40, 42, 54, 55, 61, 62, 71, 72, 81, 83, 84, and 89) HPV types. Of these, 8 HPV DNA types (11, 26, 55, 64, 69, 71, 72, and IS39) were not detected in any of the samples (**Table 3**). HPV40 and 82 DNA were each detected in only one participant. HPV6, 33, and 67 DNA were each detected in only two participants.

**Table 3:** HPV types detected in cervical cytology using the linear array HPV genotyping assay.

HPV Type	Normal Pap with any of 4 HR types (HPV 16, 18, 33, 45) (N=41)	Abnormal Pap			Total (N=95)
		with ASCUS, no CIN (N=41)	Referred for colposcopy (N=13)	Subtotal (N=54)	
6	2 (4.9%)	0 (0%)	0 (0%)	0 (0%)	2 (2.1%)
11	0 (0.0%)	0 (0.0%)	0 (0.0%)	0 (0.0%)	0 (0.0%)
16*	26 (63.4%)	3 (7.3%)	6 (46.2%)	9 (16.7%)	35 (36.8%)
18*	9 (22%)	2 (4.9%)	0 (0%)	2 (3.7%)	11 (11.6%)
31*	1 (2.4%)	1 (2.4%)	1 (7.7%)	2 (3.7%)	3 (3.2%)
33*	0 (0%)	2 (4.9%)	0 (0%)	2 (3.7%)	2 (2.1%)
35*	4 (9.8%)	0 (0%)	0 (0%)	0 (0%)	4 (4.2%)
39*	5 (12.2%)	1 (2.4%)	1 (7.7%)	2 (3.7%)	7 (7.4%)
40	0 (0%)	0 (0%)	1 (7.7%)	1 (1.9%)	1 (1.1%)
42	2 (4.9%)	2 (4.9%)	0 (0%)	2 (3.7%)	4 (4.2%)
45*	5 (12.2%)	0 (0%)	0 (0%)	0 (0%)	5 (5.3%)
51*	2 (4.9%)	2 (4.9%)	2 (15.4%)	4 (7.4%)	6 (6.3%)
52*	3 (7.3%)	1 (2.4%)	2 (15.4%)	3 (5.6%)	6 (6.3%)
53*	3 (7.3%)	3 (7.3%)	0 (0%)	3 (5.6%)	6 (6.3%)
54	1 (2.4%)	3 (7.3%)	2 (15.4%)	5 (9.3%)	6 (6.3%)
56*	3 (7.3%)	1 (2.4%)	1 (7.7%)	2 (3.7%)	5 (5.3%)
58*	1 (2.4%)	0 (0%)	2 (15.4%)	2 (3.7%)	3 (3.2%)
59*	4 (9.8%)	2 (4.9%)	0 (0%)	2 (3.7%)	6 (6.3%)
61	1 (2.4%)	2 (4.9%)	0 (0%)	2 (3.7%)	3 (3.2%)
62	3 (7.3%)	4 (9.8%)	0 (0%)	4 (7.4%)	7 (7.4%)
66*	1 (2.4%)	1 (2.4%)	2 (15.4%)	3 (5.6%)	4 (4.2%)
67*	2 (4.9%)	0 (0%)	0 (0%)	0 (0%)	2 (2.1%)
68*	3 (7.3%)	2 (4.9%)	1 (7.7%)	3 (5.6%)	6 (6.3%)
70*	3 (7.3%)	1 (2.4%)	0 (0%)	1 (1.9%)	4 (4.2%)
73*	2 (4.9%)	1 (2.4%)	0 (0%)	1 (2.9%)	3 (3.2%)
81	2 (4.9%)	1 (2.4%)	0 (0%)	1 (1.9%)	3 (3.2%)
82*	1 (2.4%)	0 (0%)	0 (0%)	0 (0%)	1 (1.1%)
83	1 (2.4%)	3 (7.3%)	0 (0%)	3 (5.6%)	4 (4.2%)
84	3 (7.3%)	3 (7.3%)	0 (0%)	3 (5.6%)	6 (6.3%)
89	3 (7.3%)	0 (0%)	2 (15.4%)	2 (3.7%)	5 (5.3%)
No HPV	0 (0%)	19 (46.3%)	2 (15.4%)	21 (38.9%)	21 (15.4%)
Any HR	41 (100%)	18 (43.9%)	11 (84.6%)	29 (53.7%)	70 (51.5%)
Any LR	13 (31.7%)	11 (26.8%)	5 (38.5%)	16 (29.6%)	29 (21.3%)

*High-risk HPV type.

HPV 26, 55, 64, 69, 71, 72, and IS39 were not detected in any of the samples included in the study and specific Abs to these types were not investigated.

For all study participants, there was no co-infection with more than one of the 4 HR types – HPV16, 18, 33, and 45. The majority (63.4%) of women with normal cytology and one of the 4 HR types had infections with multiple HPV types. For women with abnormal Pap (n=54), the majority (66.7%) did not have detectable infections with multiple HPV types. The majority (63.4%) of women with normal Pap and any of 4 HR types were positive for HPV16 DNA. The second most frequently detected DNA type in these women was HPV18 (in 22% of women; **Table 3**). HPV16 DNA was also the most frequent type detected in all women with abnormal Pap (16.7%) and in almost half (46.2%) of women referred for colposcopy. The proportion of women who had any HR HPV DNA type was 53.7% among women with abnormal Pap and 51.5% among all study participants (**Table 3**). Among women with normal Pap and any of 4 HR types, 31.7% had a co-infection with at least one LR type. The proportion of women who had any LR HPV DNA type was 29.6% among women with abnormal Pap and 21.3% among all study participants.

### 3.4 Frequency of Ab response against HPV

The frequency of HPV-specific IgG Abs in serum samples from women in the four groups under study is summarized in **Table 4**. Abs to any HPV protein were detected in 47.6% (95% C.I.: 36.5-58.8%) and 40.7% (95% C.I.: 27.9-54.9%) of women with normal and abnormal cytology, respectively and in 44.9% (95% C.I.: 36.4-53.6%) of all study participants. This includes 41.5% of women who had no detectable HPV DNA in cervical cytology samples. Among women with normal Pap and any of the 4 HR HPV types 16, 18, 33, or 45, who were all HPV positive, 53.7% had HPV-specific Abs. Abs against any E protein were detected in 47.6% and 40.7% of women with normal and abnormal Pap, respectively. Abs against any L protein were detected in 39.0% and 33.3% of women with normal and abnormal Pap, respectively.

**Table 4:** Frequency of HPV protein-specific seropositivity.

Protein	N (%)						
	Normal Pap			Abnormal Pap			Total (N=136)
	No HPV detected (N=41)	With any of 3 HR types (HPV 16, 18, 45) (N=41)	Subtotal (N=82)	With ASCUS, no CIN (N=41)	Referred for colposcopy (N=13)	Subtotal (N=54)	
E1	11 (26.8%)	11 (26.8%)	22 (26.8%)	9 (22%)	2 (15.4%)	11 (20.4%)	33 (24.3%)
E2	10 (24.4%)	7 (17.1%)	17 (20.7%)	5 (12.2%)	1 (7.7%)	6 (11.1%)	23 (16.9%)
E4	5 (12.2%)	7 (17.1%)	12 (14.6%)	6 (14.6%)	0 (0%)	6 (11.1%)	18 (13.2%)
E5	9 (22%)	6 (14.6%)	15 (18.3%)	10 (24.4%)	1 (7.7%)	11 (20.4%)	26 (19.1%)
E6	7 (17.1%)	7 (17.1%)	14 (17.1%)	6 (14.6%)	1 (7.7%)	7 (13.0%)	21 (15.4%)
E7	11 (26.8%)	6 (14.6%)	17 (20.7%)	6 (14.6%)	0 (0%)	6 (11.1%)	23 (16.9%)
Any E	**17 (41.5%)**	**22 (53.7%)**	**39 (47.6%)**	**17 (41.5%)**	**5 (38.5%)**	**22 (40.7%)**	**61 (44.9%)**
L1	13 (31.7%)	13 (31.7%)	26 (31.7%)	14 (34.1%)	1 (7.7%)	15 (27.8%)	41 (30.1%)
L2	9 (22%)	6 (14.6%)	15 (18.3%)	9 (22%)	3 (23.1%)	12 (22.2%)	27 (19.9%)
Any L	**15 (36.6%)**	**17 (41.5%)**	**32 (39.0%)**	**17 (41.5%)**	**1 (7.7%)**	**18 (33.3%)**	**50 (36.8%)**
Any HPV protein	**17 (41.5%)**	**22 (53.7%)**	**39 (47.6%)**	**17 (41.5%)**	**5 (38.5%)**	**22 (40.7%)**	**61 (44.9%)**

Abs to any E, L, or any HPV protein are indicated in bold.

The most frequently detected Abs were against L1, detected in 30.1% (95% C.I.: 22.7-38.7%) of all study participants (in 31.7%, 95% C.I.: 22.1-43.0% and 27.8%, 95% C.I.: 16.9-41.9% of women with normal and abnormal Pap, respectively). The most frequently detected Abs against an E protein were against E1, detected in 24.3%, 95% C.I.: 17.5-32.5%) of all study participants (in 26.8% and 20.4% of women with normal and abnormal Pap, respectively). The least frequently detected Abs were against the E4 protein, detected in 13.2%, 95% C.I.: 8.2-20.4%) of all study participants (in 14.6% and 11.1% of women with normal and abnormal Pap, respectively). This was followed by Abs against E6, E7, and E2 (detected in 15.4%, 16.9%, and 16.9% of all women in the study, respectively). Two of the four women with CIN III had HPV-specific Abs. One (with HGSIL) had Abs against HPV52 E1 and the other (with squamous cell carcinoma) had Abs only to HPV31 L2 and no Abs against any of the E proteins. We found no significant difference in the Abs against the positive control proteins (**Supplementary Figure 1**) or the negative control proteins (**Supplementary Figure 2**) between women in the four groups in this study.

### 3.5 HPV type-specific Ab response

Most women who had Abs against a specific HPV type did not have type-specific HPV DNA in cervical cytology. Of all study participants, there were 6 women who showed correlation between HPV DNA in cervical cytology and Ab seropositivity. Of these, 3 had HPV16 DNA, and each of the other three had one of the three HPV DNA types 39, 45, or 58 in cervical cytology (**Table 5**). Abs were most frequently detected against HPV51 and 52 (each in 20.6% of study participants). Even though HPV16 DNA was the most frequently detected type in cervical cytology (in 36.8%, 95% C.I.: 27.4-47.4% of study participants; **Table 3**), Abs against HPV16 were remarkably the least frequently detected in serum (in 7.4%, 95% C.I.: 3.8-13.5% of study participants; **Table 5**). Most (26/35; **Table 3**) women with HPV16 DNA in cervical cytology were in the normal Pap group, and only 9.8% of them had Abs against HPV16 in serum (**Table 5**). Abs against the two LR types included on the arrays (HPV6 and 11) were detected in sera of 19.9% and 17.6% of study participants, respectively.

**Table 5:** HPV type-specific seropositivity.

HPV Serotype	Normal Pap (N=82)		Abnormal Pap (N=54)		Total (N=136)	
	With no HPV detected (N=41)	With any of 3 HR types (HPV 16, 18, 45) (N=41)	With ASCUS (N=41)	Referred for colposcopy (N=13)	Ab+	Ab+ and DNA+
6	9 (22.0%)	9 (22.0%)	9 (22.0%)	0 (0.0%)	27 (19.9%)	0 (0.0%)
11	6 (14.6%)	10 (24.4%)	7 (17.1%)	1 (7.7%)	24 (17.6%)	0 (0.0%)
16	4 (9.8%)	3 (7.3%)	3 (7.3%)	0 (0.0%)	10 (7.4%)	3 (2.2%)
18	6 (14.6%)	4 (9.8%)	2 (4.9%)	1 (7.7%)	13 (9.6%)	0 (0.0%)
31	9 (22.0%)	6 (14.6%)	9 (22.0%)	2 (15.4%)	26 (19.1%)	0 (0.0%)
33	9 (22.0%)	6 (14.6%)	6 (14.6%)	0 (0.0%)	21 (15.4%)	0 (0.0%)
35	12 (29.3%)	5 (12.2%)	8 (19.5%)	2 (15.4%)	27 (19.9%)	0 (0.0%)
39	6 (14.6%)	9 (22.0%)	10 (24.4%)	1 (7.7%)	26 (19.1%)	1 (0.7%)
45	7 (17.1%)	7 (17.1%)	5 (12.2%)	2 (15.4%)	21 (15.4%)	1 (0.7%)
51	11 (26.8%)	8 (19.5%)	9 (22.0%)	0 (0.0%)	28 (20.6%)	0 (0.0%)
52	9 (22.0%)	5 (12.2%)	11 (26.8%)	3 (23.1%)	28 (20.6%)	0 (0.0%)
58	10 (24.4%)	9 (22.0%)	6 (14.6%)	1 (7.7%)	26 (19.1%)	1 (0.7%)

### 3.6 Cross-reactivity of Abs against homologous antigens

**Figure 3:**
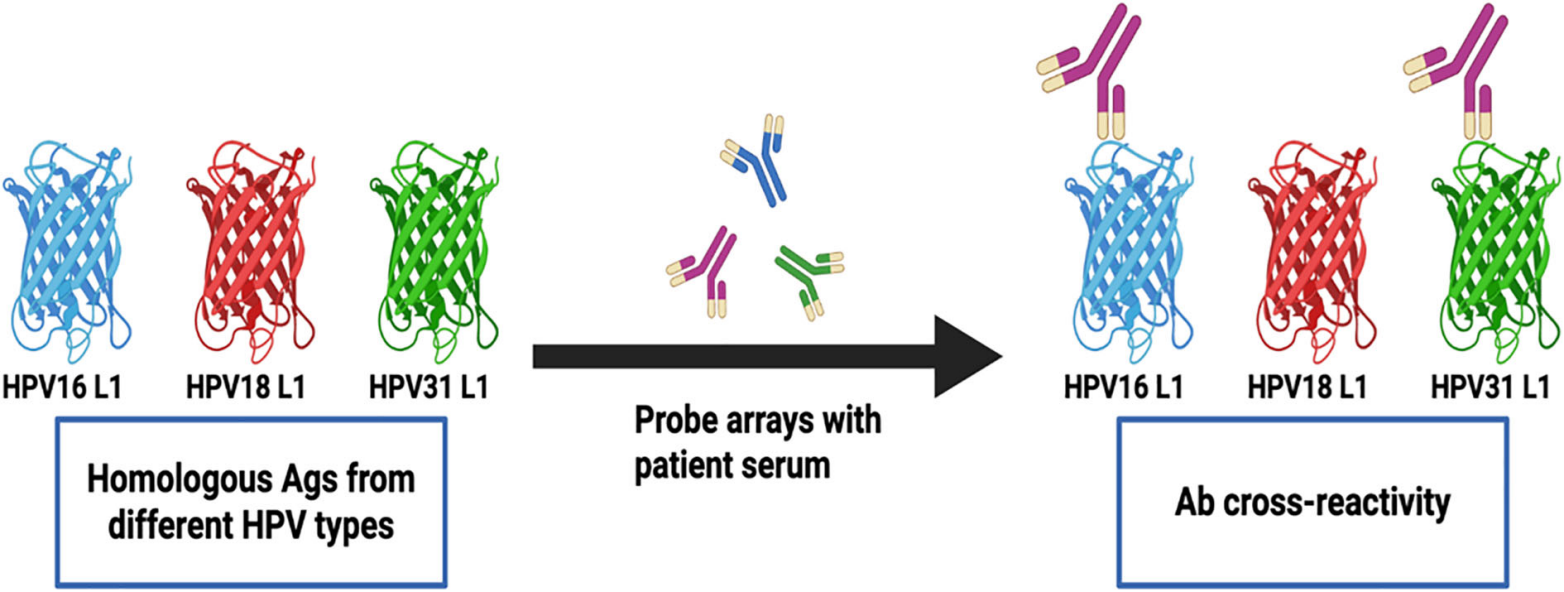
Detection of Abs against multiple homologous Ags from different HPV types. Serum Abs to a specific HPV Ag may cross-react with homologous Ags from different HPV types due to sequence similarity. The detected cross-reactivity may also be explained by past or current HPV infection with multiple HPV types in the cervix uteri or in a different anatomic site.

**Figure 4:**
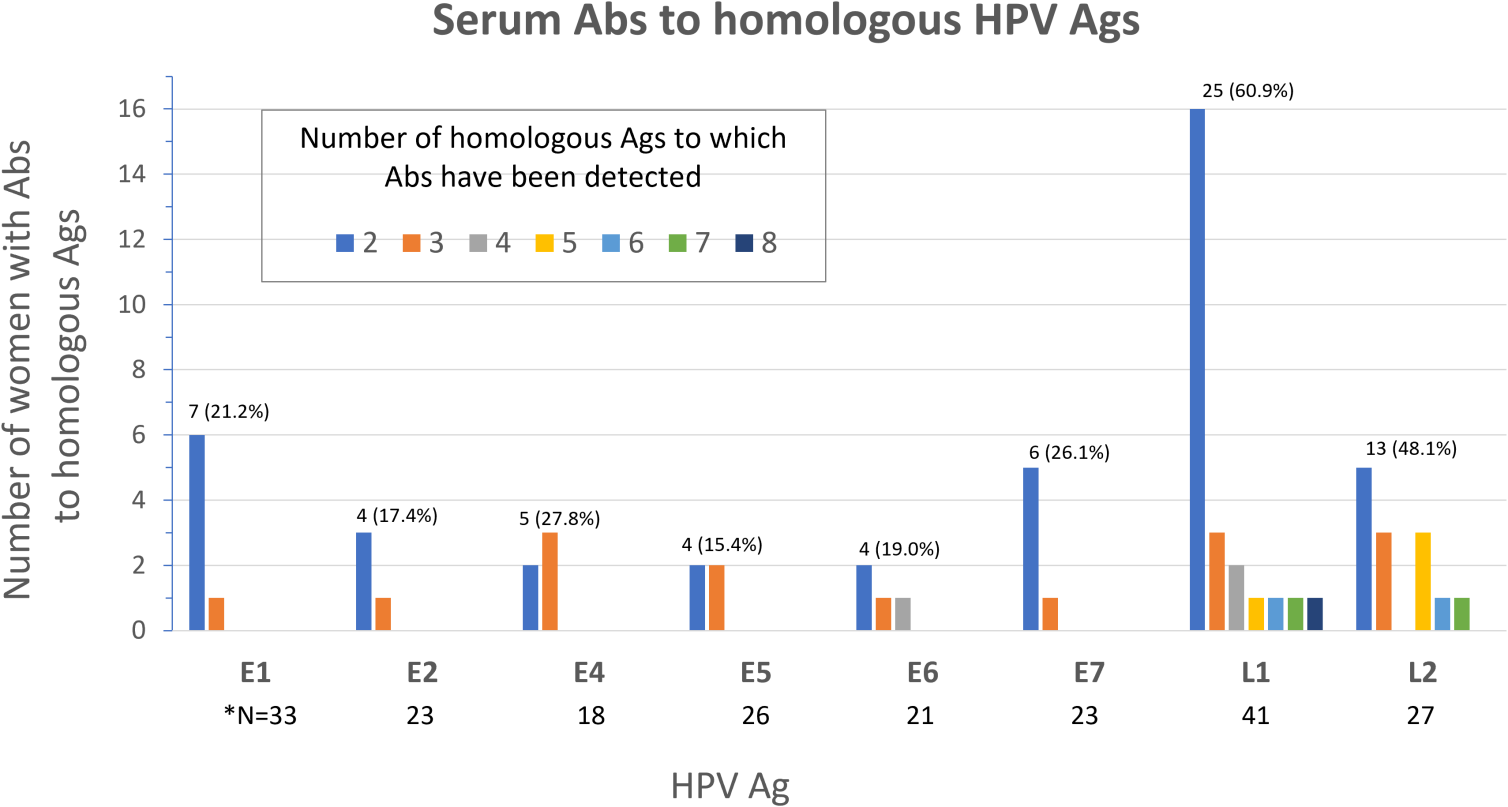
Frequency of serum Abs to homologous Ags from different HPV types among study participants (N=136). Number (%) of women with Abs to multiple (≥2) homologous Ags is shown above the bars for each Ag. *N; total number of women with Abs specific to the Ag shown.

We next sought to determine the frequency of Abs against more than one homologous Ag from different HPV types (e.g. Abs against the E1 protein from more than one HPV type in serum from the same woman) among all study participants (N=136). Homologous proteins from different HPV types have sufficient DNA and amino acid sequence similarity to allow potential binding of Abs against an HPV protein from a specific type to a homologous protein from another (Ab cross-reactivity, **Figure 3**). Abs against L1 were the most cross-reactive, followed by L2. Among women with Abs against L1 and L2, 60.9% and 48.1%, respectively showed reactivity to at least one other homologous protein (**Figure 4**). One woman had Abs against 8 different L1 proteins (from 8 different HPV types) and another against 7 different L2 proteins. The least cross-reactive were Abs against E5, with 15.4% of women with E5 Abs showing reactivity against more than one homologous E5 protein. The percentages of women with Abs that reacted to at least two homologous HPV E proteins were: 21.2% (for E1), 17.4% (E2), 27.8% (E4), 15.4% (E5), 19.0% (E6), and 26.1% (E7) (**Figure 4**).

## 4 Discussion

Serological studies have helped shape our understanding of the natural history of HPV infection and the biology of the associated carcinogenesis (55). They have also been important for evaluating the efficacy of vaccination (56) and for developing biomarkers for early detection (57). However, these studies have been limited by the diversity of over 200 HPV types and the challenges of high throughput protein expression and display. Most studies have thus focused on select HPV Ags from the most common viral types for women with invasive disease or precancerous lesions (58–60). For healthy women undergoing cervical cancer screening and for which colposcopy referral is not recommended, studies have been infrequent (57), mainly focusing on Abs to the late (L) proteins (60, 61). In this study, we have generated custom HPV HD-NAPPA arrays, displaying the proteomes of two LR and ten HR types to profile the humoral immune response in healthy women with normal or abnormal cervical cytology undergoing cancer screening. These nanowell arrays diminish the diffusion of expressed proteins to neighboring wells, allowing high throughput detection of Abs at a high analytical sensitivity (25, 26, 39). To our knowledge, this is the first study that reports comprehensive profiling of antibody response to 12 HPV proteomes in a cohort of healthy women undergoing screening for cervical cancer.

We have detected HPV-specific Abs in 44.9% of all women in the study, with no significant difference between women with normal and those with abnormal cervical cytology. This is not unexpected since HPV-specific Abs have been detected in women at different stages of HPV infection and with different types of cervical lesions (50, 55, 57, 62, 63). We have detected HPV-specific Abs in 41.5% of women who have no detectable HPV DNA in the cervix. This immune response could be against a past HPV infection that has been cleared, or an infection in a different anatomic site. Different studies have reported a wide range of frequency of HPV-specific seroreactivity in healthy controls (0-52%) (58, 64, 65) depending on the assay platform, the protein expression and display technique, and the antigen investigated (reviewed in (57)).

In this study, the most frequently detected Abs were against the L1 protein from any HPV type. They were detected in 31.7%, 27.8%, and 30.1% of women with normal cytology, women with abnormal cytology, and all women included in the study, respectively. Several studies have reported higher L1 seropositivity rates in patients with low-grade than with high-grade cervical lesions (63, 65). Using different assay platforms, they have been detected in 3-52% of healthy women (58, 59, 64, 66), likely due to a previous infection or vaccination. In cervical cancer patients, they may be a prognostic marker of better overall survival (67, 68).

Abs against E4 were the least frequently detected Abs (13.2%), followed by Abs against E6, E7, and E2 (in 15.4%, 16.9%, and 16.9% of all women in the study, respectively). E4 plays a role in viral protein synthesis and its expression correlates with viral replication, increasing in high grade lesions and dropping in invasive cervical cancer (ICC) (69). Several studies have reported increased E4 seroreactivity in women with precancerous lesions than in women with ICC (59, 60). In healthy control women, seropositivity has been detected at low frequencies (4-24%) (59, 70, 71). E4 is thus one of the most studied HPV proteins and both tissue expression and specific Abs have been proposed as early detection markers (57, 71, 72).

Because of the well-recognized oncogenic role of E6 and E7 through binding of the tumor suppressor p53 and the retinoblastoma gene product (pRb) (55, 73), Abs against these two proteins have been the most frequently investigated. They are generally more prevalent in women with invasive disease (12-54% and 13-63%, respectively) (74–78) and precancerous cervical lesions (2-14% and 6-70%, respectively) (65, 70, 79, 80) than in healthy controls (0-6% and 0-31%, respectively), correlating with disease progression. Using custom HPV NAPPA arrays, we have previously reported Abs against E7 in 30.3% of women with ICC and in 36.4% of those with ICC who were HPV16-positive (50). Both populations had advanced disease and were significantly older than the population of women in this study (mean age = 52.0 and 32.8 years, respectively), suggesting that anti-E7 Abs are more common in older age and advanced disease stage.

The most frequently detected Abs against an early protein were against E1 (in 24.3%) followed by E5 (in 19.1% of all women in the study). E1 and E5 are two of the least studied HPV proteins, especially from non-HPV16 and 18 types (57). E1-specific Abs have been reported in 0.3-4% of healthy women (59, 60). This low rate of detection could be explained by the relatively large size of the E1 protein and the challenges of expression and display of its native form and conformational epitopes. Anti-E5 Abs are not known to correlate with disease stage (57). Our lab and others have not found significant difference between anti-E5 Abs from multiple HPV types in women with ICC and precancerous lesions (50, 78).

We have detected HPV type-specific Abs in all groups of women in this study, including women with no detectable HPV DNA in the cervix. Remarkably, the least frequent type-specific Abs were against HPV16, which were detected in 9.8%, 7.3%, 7.3%, and 0.0% in the four groups of women, respectively. Only 3 women in the study (2.2% of HPV-positive women) had both HPV16 DNA in the cervix and anti-HPV16 Abs. This is not unexpected in this population of women with mostly either normal cytology or less advanced disease. We have previously reported anti-HPV-16 Ab frequencies of 6.6%, 17.1%, and 35.5% in women with CIN 0/I, CIN II/III, and ICC, respectively (50). This had coincided with HPV16 DNA detection in the cervix in 0.0%, 78.6%, and 37.0% of the same groups, respectively (50). Taken together, these data suggest that Abs against HPV16, especially with persistence of HPV16 DNA in the cervix, are more prevalent with advanced disease stage. The vast majority of women in the study did not have both HPV DNA in cervical cells and Abs from the same HPV type. This may indicate that most of the serological response detected reflects past infections that have been cleared or cross-reactivity with closely related HPV types.

Consistent with findings from our group (49, 50) and others (78), here we have detected Abs against homologous Ags from multiple HPV types most notably in 60.9% and 48.1% of women for L1- and L2-specific Abs. We have detected Abs against 8 different L1 proteins in one woman and to 7 different L2 proteins in another. In these two women, the HPV types detected in cervical cytology DNA testing were HPV 53 and 70 in the former and HPV 16 and 66 in the latter. Antibodies to three of these four HPV types are not being detected in our assay. Additionally, most (67.6%) women in this study had either no or one HPV type detected in cervical cytology DNA testing. Taken together, seroreactivity to homologous HPV proteins that we report could reflect potential Ab cross-reactivity due to sequence similarity, although current or past infection with multiple HPV types cannot be excluded. L1 and L2 are two structural proteins that form the viral capsid (81). The HPV capsids were demonstrated to contain type-common antigenic epitopes, allowing cross-reactivity between different HPV types (82). L2 also shows high sequence conservation across different types (83). Cross-reactivity may represent a challenge in serologic biomarker development for early detection of cervical cancer.

Further studies are needed before HPV-specific antibodies can be used for early detection of cancer in the general population or in a population of women undergoing cervical cancer screening. Although seropositivity rates increase with cervical disease progression, they were still detectable in our study in women with normal cervical cytology with no HPV DNA. Seropositivity may alternatively be used in low-resource settings for selection of high-risk patients for further clinical follow-up. L1- antibodies induced by vaccination against HPV are known to confer protection against HPV infection. However, further studies are needed to establish the extent of protection provided by antibodies naturally produced by HPV infection, although at least short-term protection against some HPV types has been demonstrated (84, 85).

One limitation of our study is the small sample size of women referred for colposcopy (N=13) and women with cervical cancer (N=1), which is expected for a population of women undergoing routine screening for cervical cancer in a high-income country. Because seroconversion rates in cervical cancer are in the range of 50-70% (86), it is difficult to draw generalizable conclusions from this small sample size of women whose abnormal cytology requires further investigation or intervention. Most women with abnormal cervical cytology in this study were in the early stages of disease and show lower rates of seropositivity than women with more advanced disease. Another limitation is the unknown vaccination status for most participants. Since the available vaccines induce the production of anti-L1 Abs, only our results for Abs specific for the L1 Ag are likely to be impacted by vaccination status. We were also limited by the availability of a single serum sample from each patient at the time of Pap smear collection. A longitudinal study that follows patients over time would help shape our understanding of the evolution of the humoral immune response in early HPV infection and cervical disease. A known limitation of HPV seroprevalence studies is the arbitrary nature of the cut-off value used for defining seropositivity, which makes it difficult to directly compare the results of different studies (57, 87).

In conclusion, we report serum immune profiles of women undergoing screening for cervical cancer to 12 HPV proteomes. Our results have important implications for the use of serology for early detection, vaccine development, and understanding of the virus biology and pathogenesis. There is great interest in serology for early biomarkers of cervical disease, especially in low- and middle-income countries and for point-of-care testing to address limited resources available for screening (88, 89). For serology to be used as a reliable marker for early detection, typing, or selection of patients for colposcopy, better understanding of HPV-specific immune response is needed. More studies are needed on women undergoing screening for cervical cancer, especially those with abnormal cervical cytology to help inform our understanding of the immune response. Up to 80% of patients with HPV-positive oropharyngeal cancer have Abs against at least one early Ag (90–92), reflecting potentially different underlying virus biology in these two sites and the challenges of using serology for cervical disease. Our findings shed light on the kinetics of HPV-specific humoral immunity in women with normal or abnormal cervical cytology and highlight the need for comprehensive immune profiling in different health and disease stages.

## Data Availability

The original contributions presented in the study are included in the article/**Supplementary Material**, further inquiries can be directed to the corresponding author.

## Supplementary material

Supplementary Figure 1 Heatmap showing Ab seroreactivity to positive control proteins among women in the four study groups and patients with HPV-associated oropharyngeal cancer (HPVOPC). EBNA-1, Epstein-Barr Nuclear Antigen-1; EBNA-2, Epstein-Barr Nuclear Antigen-2; EBV BFRF3, Epstein-Barr virus small capsomere-interacting protein; H1N1-Np, H1N1 Nucleoprotein; H3N2-Np, H3N2 Nucleoprotein; UL122, HCMV2 Viral transcription factor IE2. Darker color indicates higher seroreactivity.

Supplementary Figure 2 Heatmap showing Ab seroreactivity to negative control proteins among women in the four study groups and patients with HPV-associated oropharyngeal cancer (HPVOPC). Darker color indicates higher seroreactivity. Negative controls included 93 genes (including the plasmid encoding the GST fusion protein) and printing master mix (MM) with no plasmid.

Supplementary Table 1 Raw signal intensity data obtained from all high-density nanowell arrays used in this study.
