# Supplementary figures and images for: Serum profiling of the antibody response to HPV in women with or without abnormal cervical cytology undergoing cervical cancer screening
Source: Front Immunol. 2025 Jul 31;16:1612761. doi: 10.3389/fimmu.2025.1612761 (PMC12350350; doi:10.3389/fimmu.2025.1612761)

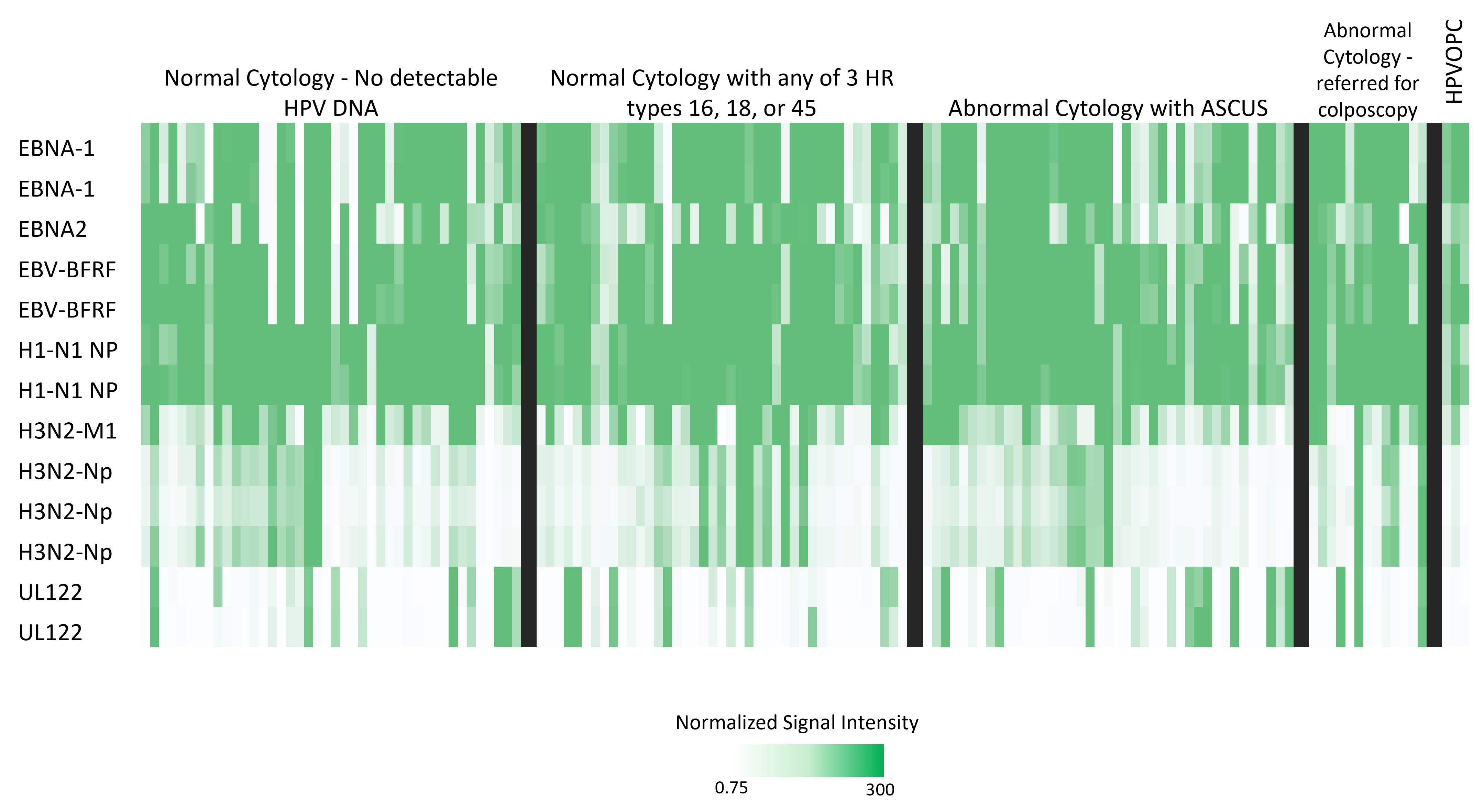

Supplement: Supplementary Figure 1 — Heatmap showing Ab seroreactivity to positive control proteins among women in the four study groups and patients with HPV-associated oropharyngeal cancer (HPVOPC). EBNA-1, Epstein-Barr Nuclear Antigen-1; EBNA-2, Epstein-Barr Nuclear Antigen-2; EBV BFRF3, Epstein-Barr virus small capsomere-interacting protein; H1N1-Np, H1N1 Nucleoprotein; H3N2-Np, H3N2 Nucleoprotein; UL122, HCMV2 Viral transcription factor IE2. Darker color indicates higher seroreactivity. [file Image1.tif]

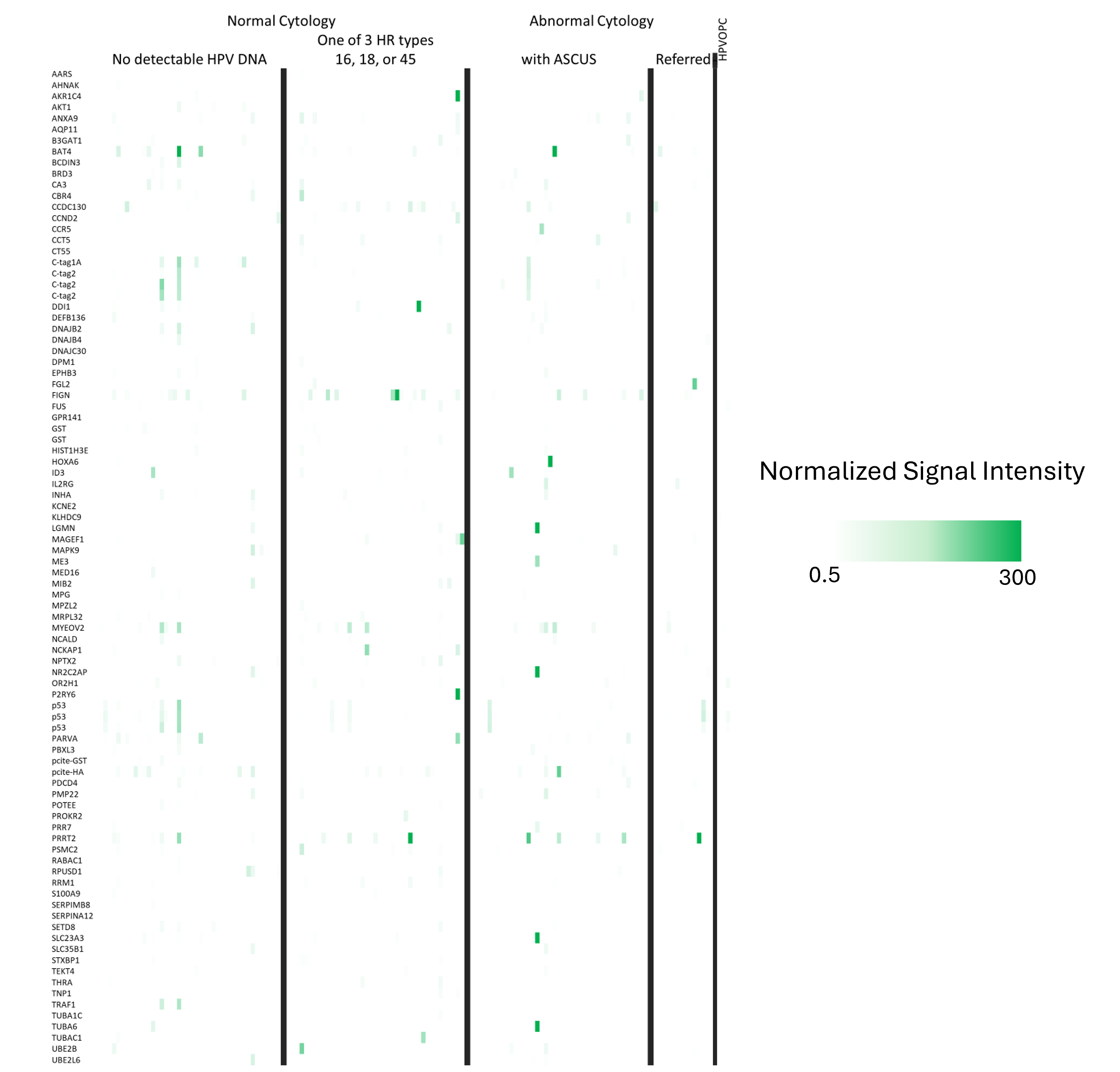

Supplement: Supplementary Figure 2 — Heatmap showing Ab seroreactivity to negative control proteins among women in the four study groups and patients with HPV-associated oropharyngeal cancer (HPVOPC). Darker color indicates higher seroreactivity. Negative controls included 93 genes (including the plasmid encoding the GST fusion protein) and printing master mix (MM) with no plasmid. [file Image2.tif]
